# Attachment Classification, Emotion Regulation, and Defense Mechanisms: An Integrative Narrative Review

**DOI:** 10.3390/healthcare13233105

**Published:** 2025-11-28

**Authors:** Arielle M. Morris, Emma Freeberg-Powell, Shivani Verma, Mariagrazia Di Giuseppe, Hunter Crespo, Leon Hoffman, Timothy Rice

**Affiliations:** 1 College of Medicine, Downstate Health Sciences University, New York, NY 11203, USA; arielle.morris@downstate.edu; 2 Silberman School of Social Work, Hunter College, New York, NY 10035, USA; epowell@wesleyan.edu; 3 Barnard College, Columbia University, New York, NY 10027, USA; sv2807@barnard.edu; 4 Department of History, Humanities and Society, University of Rome Tor Vergata, 00133 Rome, Italy; 5 Department of Psychology, The New School for Social Research, New York, NY 10003, USA; cresh450@newschool.edu; 6 New York Psychoanalytic Society and Institute, New York, NY 10028, USA; hoffman.leon@gmail.com; 7 Department of Psychiatry, Icahn School of Medicine at Mount Sinai, New York, NY 10029, USA; 8 Department of Psychiatry, Columbia University Vagelos College of Physicians and Surgeons, New York, NY 10032, USA

**Keywords:** attachment, emotion regulation, defense mechanisms, assessment, social determinants of health, clinical interventions

## Abstract

**Highlights:**

**What are the main findings?**

- Attachment styles are strongly linked to emotion regulation and specific defense mechanisms across the lifespan.
- Understanding a patient’s defensive patterns offers a window into their underlying attachment style in clinical settings.

**What are the implications of the main findings?**

- Clinicians can individually tailor interventions by integrating attachment, defenses, and emotion regulation into assessment and treatment.
- A defense-informed approach provides clinicians with deeper insight into patients’ psychological functioning and supports the development of comprehensive, individualized therapeutic strategies.

**Abstract:**

Attachment style and emotion regulation (ER) patterns intertwine. Securely attached individuals employ more adaptive ER strategies, while individuals with avoidant, preoccupied, and disorganized styles rely on less adaptive strategies. Defense mechanisms are part of an experience-near, observable construct that parallels implicit ER. The evaluation of a patient’s defense mechanisms may therefore be a means of identifying and understanding the patient’s attachment classification. This article synthesizes recent empirical research and theory to delineate relationships among attachment styles, ER, and defense mechanisms. It then examines how development and culture shape attachment, discusses assessment strategies, and offers clinicians guidance for assessing attachment through a defense mechanism orientation. This clinical technique may assist clinicians in informed assessment and treatment and underscores the benefits of further integration of attachment research with that of defense mechanisms.

## 1. Introduction

Attachment theory provides a foundational framework for understanding how early relational experiences shape psychological functioning and psychopathology across the lifespan [1,2]. Bowlby’s concept of internal working models of self and other explains how early caregiver–child bonds create expectations for safety, trust, and self-worth, as well as their downstream effects on relational and emotional regulation capacities [3,4,5,6,7]. Ainsworth and colleagues [8,9,10] operationalized these ideas in infant–mother dyads, identifying secure, anxious–ambivalent, and avoidant attachment patterns, later expanded to include disorganized–fearful attachment [11]. Subsequent research extended these categories to adulthood, first in romantic relationships, identifying secure, avoidant, and anxious–ambivalent styles [12], later refining them into secure, anxious–preoccupied, dismissing–avoidant, and fearful–avoidant or disorganized styles [13]. These adult presentations correspond to childhood attachment patterns. In particular, insecure attachments in infancy—specifically disorganized—are consistently associated with greater motivation to maintain emotional distance and minimize attachment needs in adulthood [7].

Across the lifespan, secure attachment is consistently associated with more adaptive emotion regulation (ER)—the capacity to adjust one’s emotional responses to adapt to situational demands [14], whereas insecure patterns accompany vulnerabilities for regulatory difficulties and long-term psychopathology [11,14,15,16]. Cassidy [17] conceptualized ER as originating within the attachment relationship itself, proposing that infants develop strategies, rooted in internal working models, to regulate affect in ways that maintain proximity to and support from caregivers. Building on Thompson’s definition [18], she emphasized that these regulatory processes involve both intrinsic and extrinsic factors, through which children learn to suppress or heighten emotional expression to preserve attachment security. Adults with secure attachment demonstrate greater use of more adaptive ER strategies, such as cognitive reappraisal, compared to their insecurely attached counterparts [16,19], who tend to use less adaptive ER strategies, including suppression.

Beyond the parallels between attachment and ER, this review further integrates these constructs with defense mechanisms—involuntary mental processes that modulate individual responses to impulses, affects, and thoughts, during stress and contribute to psychological stability [20,21]. We propose that observing defense mechanisms as they occur in clinical encounters offers a valuable lens for understanding a patient’s attachment style and ER capacities. We synthesize theoretical and empirical research linking attachment, ER, and defenses. Further, we consider the impact of development and cultural influences, review current assessment approaches, and offer guidance for clinicians in the assessment of individuals’ attachment styles through a defense and regulatory orientation. To develop our understanding of the relationships between these concepts, we conducted a literature review with the method outlined in Table 1.

## 2. Implicit ER and Defense Mechanisms

Implicit ER and defense mechanisms are conceptually and functionally interconnected processes for modulating affect outside of conscious awareness. Gross’s process model outlines explicit ER—conscious, deliberate adjustment of emotional responses to meet situational demands, such as stressors [22,23]. In contrast, implicit ER operates outside of conscious awareness to maintain emotional stability [24]. When defenses are conceptualized as mechanisms for the protection of the individual from painful or threatening emotions, defenses conceptually align with implicit ER, consistent with the ego-psychological views of defenses as unconscious operations [25]. This alignment provides a shared framework, linking neuroscientific ER literature with psychodynamic accounts of defenses.

Vaillant’s [8,9] hierarchical model of defense mechanisms categorizes defenses along a continuum from immature or pathological (e.g., acting out, passive aggression, denial, splitting) to moderately neurotic (e.g., dissociation, intellectualization, displacement) to mature or adaptive (e.g., self-observation, anticipation, suppression, affiliation, humor) [26,27]. This gradient mirrors the continuum of the observed hierarchy of adaptiveness in the ER literature, linking more adaptive responses to better psychological functioning [24,25].

From a developmental and neurobiological perspective, ER capacities rely on prefrontally-mediated modulation of limbic activity. Psychoanalyst Berta Bornstein’s formulation of children’s defenses as operations against painful affect [14] provides a pathway to understand the similarity of these two constructs. Within the dual-process framework of Gyurak and Etkin [24], implicit and explicit emotion regulation map onto distinct neural systems. Implicit ER comprises automatic adjustments in self and environmental evaluations with immediate response tendencies to emotional stimuli, and has defined neural correlates within the ventral prefrontal cortex (vPFC). In contrast, explicit ER is functionally distinct, originating in more dorsolateral regions of the prefrontal cortex (dlPFC), and is characterized by conscious reappraisal, distraction, or suppression of emotional responses. Mapping implicit (vPFC) and explicit (dlPFC) regulation onto distinct systems clarifies why avoidantly attached profiles, organized around deactivating regulatory strategies and reduced conscious affective evaluation of threat, and anxiously attached profiles, associated with hyperactivating strategies and heightened conscious affective evaluation of threat, diverge in their defensive operations and in their characteristic use of suppression or cognitive reappraisal. This provides a rationale for differentiating ER strategies by attachment profile in assessment and treatment.

## 3. Existing Studies of Attachment Style, ER, and Defense Mechanisms

Several empirical studies demonstrate the link between attachment style, emotion regulation, and defenses. In psychiatrically hospitalized adolescents, fearful attachment was linked to difficulties describing emotions and reliance on the most immature defenses, whereas anxious and preoccupied attachment styles were associated with challenges identifying emotions and a combination of immature and neurotic defenses [4]. These findings suggest generally lower ER capacities among non-secure attachment profiles, alongside distinct defensive tendencies across insecure styles during adolescence. In depressive adult patients, secure attachment corresponded with higher implicit ER functioning and more mature defenses, while insecure attachment was tied to less adaptive ER functioning and more immature defenses [5]. Together, these findings highlight how attachment patterns shape both explicit and implicit regulatory processes, offering clinically meaningful markers for assessment and intervention.

## 4. Clinical Applications: Integrated Attachment, Emotion Regulation, and Defense Mechanisms

Empirical work reinforces the interrelatedness of ER within the context of attachment. A recent systematic review examined attachment style and ER in adults [7]. The authors found that research consistently supports a clear correlation between attachment style and ER, where attachment security is associated with effective ER, and insecure or disorganized–unresolved attachment is associated with dysfunctional ER.

A substantial body of research links ER strategies, as well as defense mechanisms, to distinct attachment profiles across the lifespan. Because these patterns emerge early and consolidate over time, treatment can be tailored to each patient’s unique presentation. Below, we describe patients’ attachment profiles and their associated ER and defense strategies and summarize the broad categorical differences.

### 4.1. Secure Attachment

From infancy through older adulthood, secure attachment is associated with adaptive ER strategies and mature defense mechanisms. Specifically, secure attachment has been linked to balanced regulation of one’s emotions, high self-esteem, productive support-seeking, consistently effective cognitive reappraisal (reinterpreting an emotional situation to reduce negative feelings and arousal), and a minimal reliance on suppression [7,19,28]. In infancy and childhood, secure individuals rely on their caregivers as secure bases for soothing, exploration, and emotional stability [29]. In adolescence, they employ strong cognitive reappraisal and, when appropriate, suppression to regulate their emotions, with the expectation that their emotional responses will be received appropriately [30]. In adulthood, securely attached individuals have a strong capacity to regulate emotions, particularly with regard to cognitive reappraisal, which is biologically indicated with high prefrontal cortex modulation of the amygdala during ER activities [19,31,32]. Secure adults also show a high capacity for reflective functioning or mentalization, as well as adaptive and mature defenses [5], and exhibit successful psychosocial adjustment to changes presented even in later life [33]. Treatment for securely attached individuals may support a high willingness to engage in treatment, building on adaptive strengths already present, further deepening insights, and promoting growth during challenging life experiences [7,34].

### 4.2. Anxious, Ambivalent, or Preoccupied Attachment

Anxious–preoccupied attachment employs hyperactivating strategies (e.g., rumination, emotional intensification, compulsive caregiving, proximity-seeking), used to secure reassurance, but often perpetuate chronic distress [3,7,35,36,37,38,39]. Beginning in infancy and childhood, these individuals display intense emotional reactions, even in the presence of an attachment figure, and difficulty with soothing after separation [29,37]. In adolescence, these presentations often reflect earlier unpredictable caregiver responses to support-seeking in childhood [14,40]. Across adolescence, they show marked emotional dysregulation of sadness, anger, and worry, along with suppression of anger [40,41]. From adolescence into adulthood, anxiously attached individuals frequently rely on immature depressive and neurotic defenses (e.g., splitting, projective identification, acting out, passive aggression, reaction formation, undoing, hypervigilance) [20,21]. In adulthood, anxious attachment correlates with certain ER strategies, including greater use of cognitive reappraisal (adaptive) as well as rumination and suppression (maladaptive) [38]. In bereavement later in life, anxious adults maintain hypervigilance to painful reminders of their loss and struggle to suppress attachment-related pining when faced with the death of a partner [42]. Given that anxiously attached adults struggle to regulate high emotional arousal [19], interventions should first stabilize arousal before engaging in deeper cognitive work and then incorporate cognitive reappraisal to interrupt rumination and reduce reliance on suppression [36,38].

### 4.3. Avoidant or Dismissing Attachment

Avoidant–dismissing attachment profiles rely on deactivating emotional regulation strategies (e.g., suppression, emotional distancing, denial), which serve to minimize distress, maintain self-reliance, and resist vulnerability [3,7,35,43,44]. Infants and young children who experience consistent negative responses to their support-seeking tend to develop avoidant/dismissing attachment profiles [41,45,46]. Early in life, children minimize outward signs of distress during separations from their caregivers, despite elevated physiological arousal [41,45,46]. Avoidant adolescents continue to suppress certain emotions (sadness, worry) while showing emotion dysregulation during anger [40]. In adulthood, defensive isolation and emotional disengagement are common [41], again accompanied by high physiological arousal during emotional suppression [19]. Among bereaved older adults with avoidant attachment, self-reported yearning is lower, and acceptance is higher, yet somatic symptoms are more likely [42]. Clinical treatment should build emotional awareness and tolerance, encourage willingness to approach closeness and develop openness, and shift patients away from suppression and emotional disengagement [5,47].

### 4.4. Fearful, Unresolved, or Disorganized Attachment

Research on developmental stages and differences for fearful/disorganized attachment remains limited. However, available studies provide clinically relevant insights rather than a uniform developmental trajectory. Disorganized attachment, which often co-occurs with trauma or borderline features [48], involves difficulty tolerating and integrating overwhelming emotions [48]. They also frequently present with primitive defenses (e.g., splitting, dissociation, emotional disengagement) [49]. These patients may benefit from structured, trauma-informed approaches that educate them about the interpersonal effects of their behavior and reduce alienating behavioral expression [34]. Stabilization, mentalization-based therapy, and dialectical behavior therapy can improve emotional identification and clarity, and reduce immature defenses [48,49,50].

### 4.5. Comparison of Secure and Insecure Attachment Profiles

Across development, converging evidence links secure attachment with adaptive ER and mature defensive strategies, and ties insecure attachment with less adaptive ER and immature defenses. In adoptive familial contexts, children of securely attached parents demonstrate more organized and adaptive ER strategies during conflict tasks than children of parents with insecure attachment profiles [2]. This highlights intergenerational transmission of regulatory capacities within attachment relationships. In adolescence, secure attachment is marked by higher concordance between facial emotional expression and self-reported anger and sadness, suggesting higher regulatory capacities [15]. However, this concordance was low for insecurely attached adolescents, reflecting either emotional avoidance for dismissing–avoidantly attached adolescents or emotional over-engagement for preoccupied–anxiously attached adolescents. In adulthood, joint assessment using the Adult Attachment Interview (AAI) for attachment [51] and the Defense Mechanisms Rating Scale (DMRS) for defenses [20] demonstrates that secure attachment is positively associated with reflective functioning (mentalization) and adaptive or mature defense mechanisms (coded as implicit ER) [5]. Conversely, individuals with insecure attachment may frequently rely on immature defense mechanisms as a means of avoiding awareness of mental distress, attempting to block conscious recognition of psychological discomfort [5,25]. These findings highlight defense mechanisms as a crucial lens for understanding unconscious emotion regulation capacities across the lifespan.

Both anxiously and avoidantly attached individuals use cognitive reappraisal and suppression to reduce displeasure but are generally less effective in regulating arousal, indicating overall less adaptive ER [19]. These distinctions are particularly salient in stressful situations, where the chosen ER approach may directly shape clinical presentation and inform targeted intervention. In contrast, disorganized attachment lacks a coherent ER strategy and is associated with heightened stress reactivity, dissociation following trauma, prolonged recovery from stress, and rapid emotional fluctuations, though individuals with this profile can employ moderately effective cognitive reappraisal and suppression to regulate displeasure and arousal [7,19,52,53,54]. By recognizing and distinguishing between these attachment-informed regulation trajectories and defenses across the lifespan, clinicians can tailor patient interventions, improve a patient’s psychological functioning, and strengthen the quality of therapeutic treatment.

## 5. Developmental Considerations

Attachment and ER patterns emerge in infancy through bidirectional caregiver–infant interactions, in which each partner responds to the other’s cues, resulting in mutual co-regulation ER [55,56]. Co-regulation of emotion reflects the interplay between interactive contingency (how each partner in the caregiver-infant dyad coordinates gaze, facial affect, vocal timing/prosody, and turn-taking from moment to moment) and self-contingency (each partner’s own behavioral timing patterns), with self-processes often providing the stronger influence during dyadic exchanges, even as they are shaped by the interaction [55]. Longitudinal work shows that secure attachment in infancy is associated with more sensitive, cooperative caregiving, whereas both avoidant and resistant attachment are associated with lower caregiver sensitivity [56]. Across the sample, infant temperament alone did not reliably predict attachment security. Instead, caregiver sensitivity moderated temperament effects: caregiver sensitivity at 6 months fully mediated the impact of newborn irritability on later attachment security [56]. Disorganized attachment was best predicted by caregiver intrusiveness and maltreatment (including psychological unavailability and physical abuse), and was not explained by infant temperament [56].

Reflective functioning or mentalization (the capacity to understand one’s own and others’ internal mental states) provides a measurable bridge between attachment and ER: in clinical samples, secure attachment is associated with higher reflective functioning and overall defensive functioning, which correlate with more mature, affect-regulating defenses that support adaptive development [5].

Children continue to adapt through exposure to life events and developmental stages [15,57,58]. In adolescence, caregiver attachment shapes both general ER capacity [57,58] and ER during peer-related problem-solving tasks [15], contributing to substantial variability in ER capacities at this stage [57,58]. In adulthood, major stressors and relationship changes can alter attachment patterns [59,60] and influence ER strategies, with high stress exposure linked to maladaptive regulatory responses such as catastrophizing [61]. Later life brings adaptations in attachment needs, including fewer close relationships, greater reliance on symbolic attachments, and reduced attachment anxiety but not avoidance [62] alongside heightened vulnerability to separation and loss [63]. In older adults, avoidance, rumination, and growth-oriented coping can mediate the impact of earlier stressors on current distress, underscoring the evolving interplay between attachment, ER, and life stage [64].

Similarly, Cramer [35,65,66,67] studied developmental shifts in childhood through adolescence in the use of defense mechanisms. Reliance on major image-distorting defenses (e.g., denial and projection) gradually recedes through a shift to less impairing and more mature defenses (e.g., identification).

Taken together, these findings provide evidence to support the value of understanding the parallels between attachment, defense, and ER patterns and how they shift across developmental stages in clinical practice. Specifically, they highlight the importance of using comprehensive, multi-method, longitudinal assessment approaches to accurately capture these dynamics in clinical settings and tailor treatment to patients’ unique needs. We have summarized these parallels in Table 2.

## 6. Assessing Attachment Through Defensive Strategies and Emotion Regulation Profiles

By examining patients’ ER and defensive patterns, clinicians can gain subtle yet significant insight into patients’ underlying attachment models and development that led to their current state [3,69]. Defensive patterns, including intellectualization, projection, or dissociation, can be indicative of underlying attachment dynamics [41,49]. New measures like the Attachment Defenses Questionnaire expand assessment options by linking defenses to attachment styles [3]. Specifically, they found that defenses categorized as hyperactive, including splitting and compulsive caregiving, correlated with an anxious attachment profile [3]. In addition, this study showed that defenses characterized as deactivating, such as suppression of specific emotions like anger or efforts to maintain independence, correlated with an avoidant attachment profile [3].

There are also biological links between attachment and ER capacities. For instance, anxiously attached individuals typically show heightened physiological stress responses alongside overt emotional expression, reflecting hypervigilant strategies [70]. Individuals with insecure-dismissing attachment often outwardly present emotional detachment while showing elevated sympathetic activation, such as increased skin conductance, indicating a mismatch between self-reported emotions and physiological distress [2,15,71,72,73]. Disorganized–unresolved individuals display context-dependent avoidance motivation (an emotional dimension) at a physiological level, as evidenced by specific activation patterns in the amygdala and anterior cingulate cortex when exposed to attachment-related stressors—particularly images of an unknown crying child compared to images of their own distressed infant [2].

Moreover, event-related potentials (ERPs), such as the late positive potential (LPP), capture the cerebral processing of emotional information, especially arousing information, contributing to their common use in ER studies [74,75]. In a neurophysiological electroencephalography (EEG) study examining LPPS, higher LPP amplitudes during a reappraisal task, in response to a distressing stimulus, moderated and strengthened the relationship between attachment anxiety and ER difficulties, but this moderating effect was not present when there were lower LPP amplitudes, and there was also no moderating effect for any LPP amplitude for avoidant attachment profiles [76]. Thus, this study suggests that biomarkers indicating emotional processing abilities could be an effective tool for developing clinical formulations tailored to individuals with certain attachment styles and to develop a more holistic understanding of patients. Taken together, these studies indicate the relevance of considering both psychological and biological correlates of attachment. Integrating assessments of attachment to include defense mechanisms and ER, as this will assist in developing comprehensive clinical profiles.

Currently, primarily clinical researchers use structured interviews, projective measures, and rating scales to capture nuanced aspects of the attachment system (e.g., narrative vs. physiological), and multi-method assessment approaches allow for targeted clinical insight and application, as shown in Table 3.

Although many of the known assessment tools provide valuable insights in their respective domains, a comprehensive method that captures attachment, ER, and defense mechanisms within a single assessment framework accessible to clinicians is still lacking. Even promising instruments like the Attachment Defenses Questionnaire (ADQ-50) [3], which bridges attachment and defense strategies, fall short of integrating physiological, narrative, or behavioral indicators of ER. This gap underscores the need for more holistic assessment models. To advance both research and clinical application, future efforts should prioritize the development of multidimensional tools that account for the dynamic interplay of these constructs. Integrating patients’ developmental histories, relational patterns, and neurobiological functioning into a unified, systems-based model could enhance the precision and efficacy of psychological treatment [26].

## 7. Social Considerations and Addressing Social Context for Interventions

Social determinants of health and contextual adversity similarly shape defenses, ER, and attachment classification. Income, discrimination, and chronic stress shape both attachment [80,81] and regulatory capacities [82]. Individuals with early relational disruptions, such as children growing up in socioeconomically disadvantaged contexts [83] and those with foster care histories [84], may experience higher exposure to conditions associated with insecure or disorganized attachment, and possibly later challenges with ER, while substantial individual variability remains. On average, children in low-income contexts show higher rates of insecure attachment with their mothers and greater difficulties regulating themselves during stress [83]. In one study, educational attainment was associated with attachment anxiety, but not avoidance [80]. This study also found that within the same sample, having more children was associated with higher attachment avoidance only among men, whereas lower income was associated with higher attachment avoidance only among women [80].

Trauma and chronic adversity can shape attachment patterns [49], defensive operations [85], and ER [86]. In processing experiences of adversity, individuals may experience hyperarousal or attempt to contain heightened autonomic arousal, and therapeutic approaches should integrate the altered capacity for empathy and mentalization brought upon by such defenses [49]. Therapist regulation is also important: managing the clinician’s own emotions supports the client’s mentalization, especially during high-intensity affective contexts where both partners’ regulatory and mentalization processes interact and influence one another [87]. For adults who have experienced childhood adversity, approaches that target negative self-perceptions and rumination are beneficial [86], alongside attachment-focused work that builds awareness of trauma responses [88] and interpersonal patterns [52]. For children, symbolic play combined with guided emotion labeling and cognitive reappraisal, along with coaching caregivers to model these skills between sessions, can scaffold ER and strengthen the therapeutic alliance [89]. Importantly, attachment can reorganize despite early adversity through clinical strategies, including the Dynamic Maturational Model of Attachment and Adaptation (DMM) method of coding the AAI [88]. When adoptive [2] and biological [88] mothers move toward attachment security, their children can likewise reorganize their attachment to become more secure, reducing intergenerational transmission of trauma and promoting resilience [2,88]. Tailoring care to individuals’ lived experiences and developmental stages is essential because while early adversity can alter psychobiology [36,90], attachment styles remain amenable to relational, mentalization-informed intervention later in life [2,49,88].

Supportive caregiving environments—such as trained childcare providers—can buffer adversity-related risk for attachment difficulties in children who are underserved. Eckstein and colleagues [83] found that children from low-income backgrounds, similar to their middle-income peers, were able to form secure attachments with middle-income, professionally trained childcare providers who learned to understand and respond to their circumstances [91].

Cultural frameworks systematically shape how emotions are expressed and regulated, including display rules [92], as well as cultural differences in the frequency of utilizing suppression (e.g., interdependent cultures like East Asia tend to use more expressive suppression but incur less costs, whereas independent cultures like the United States use less suppression but incur greater costs) [93,94], but not cognitive reappraisal [93,94]. These findings may be attributed to how suppression controls outward expression of emotions, which is significantly impacted by cultural display rules and norms, while reappraisal alters internal meaning-making of emotions privately, so it is less visible and socially influenced, which may explain why there are smaller cross-cultural differences in reappraisal [93,94]. 

Moreover, cultural frameworks influence how attachment insecurity is expressed in relationships [94]. Cross-cultural studies report variation in both the prevalence and behavioral manifestations of avoidant and anxious attachment (e.g., individualistic societies like Mexico tend to have higher rates of avoidant attachment, collectivist societies like the United States tend to have higher rates of anxious attachment), consistent with differences in interdependence and independence norms and caregiving practices [94,95]. 

Accordingly, assessment should consider observed behaviors within the context of the patient’s cultural norms, display rules, and caregiving expectations [94,96]. For instance, what appears as suppression in one cultural context may be considered adaptive emotion regulation in another [92,93]. Thus, cultural context should be treated as an explanatory variable for emotion regulation and attachment in both assessment and intervention selection, not merely as background demographics [94,96].

Converging evidence from diverse clinical populations across developmental stages demonstrates consistent links between attachment patterns, ER capacities, and characteristic defensive strategies. Observed differences in defensive styles should be interpreted in context: lower-level defenses are reported more often in some groups (e.g., women, younger adults, people who have never married, and individuals with lower income or educational attainment) [97]. These patterns may reflect differential stressor exposure, cultural norms, and measurement context, rather than fixed traits. More broadly, the use and adaptiveness of particular defenses are influenced by cultural milieu, stress exposure, genetic predispositions, and individual context; for example, stressful experiences, such as breast cancer, have been linked to greater reliance on lower-level defenses [97].

Despite increasing integration of attachment and regulatory concepts into clinical models [98], empirical work that explicitly integrates attachment, ER, defensive functioning, physiological measures, and sociodemographic and cultural characteristics within a unified framework remains limited [3,5,7,36]. Advancing such convergent, essential research will improve culturally attuned, developmentally sensitive assessment and enable more precisely targeted interventions.

## 8. Discussion

This review integrates original and emerging evidence linking attachment styles, ER patterns, and defensive strategies across the lifespan. We highlight how attachment functions as an organizing system for emotional and defensive processing [19,45]. Clinical practice significantly benefits from recognizing these relationships. This allows for tailored interventions that directly address how patients process, avoid, or amplify emotional experience [5,47], including how they deal with painful emotions in clinical situations [25,36,68,90,99].

Table 4 shows how clinicians can make use of their findings to identify emotion regulation, defenses, and attachment. Clinicians can organize their observations on defenses as a window into the attachment classification and ER strategies of the patient and thereby gain valuable clinical information in formulation and treatment planning. These insights can drive individualized treatment approaches.

Taken together, our findings highlight how a strong understanding of the interconnectedness of constructs can guide decision-making in therapeutic settings. Treating attachment, emotion regulation, and defenses as an integrated system allows clinicians to infer the prevailing needs of each patient. In turn, clinicians can set the structure and pacing of the intervention accordingly. As a result, this framing allows for more tailored, individualized, and effective care to strengthen patients’ regulatory capacities and develop more mature, adaptive defenses. Moreover, because cultural display rules and contextual stressors shape both expression and interpretation of affect, this holistic approach also supports equitable decision-making by distinguishing surface presentation, informed by sociocultural context, from underlying regulation capacities. Thus, our translation of psychological constructs into tangible clinical procedures and responses, while simultaneously acknowledging subjective context, offers valuable insights for improving therapeutic practice. The practical outline for this approach is articulated across the manuscript’s tools, including Table 2 (mapping of the attachment–ER–defense relations), Table 3 (assessments), and Table 4 (clinician responses), which support consistent and strength-based clinical implementation.

### Future Directions

Future research may continue to explore how attachment, defenses, and ER evolve over time, incorporating multimodal assessments such as interviews and self-report questionnaires [5]. This may be supplemented by objective measures such as EEG, functional magnetic resonance imaging (fMRI), and eye-tracking systems to further support the relationship between attachment patterns and ER in different settings [19]. Moreover, studying how social determinants can buffer these processes, either by protecting against negative outcomes or increasing vulnerabilities, is essential for designing comprehensive interventions that account for how psychosocial factors can influence psychopathology [69,85,90].

Our narrative review approach to the study and limited methodological structure could be expanded upon in a systematic review format, outlining a stepwise process for gathering and obtaining information, criteria, and further depth for each of the cited studies. The purpose of this review was to draw connections between ER, attachment, and defenses and provide an overview for clinical purposes, rather than an in-depth, detailed analysis of each study, although that would prove beneficial for further scientific understanding and more complex studies for mediation and moderation of these factors.

As defense mechanisms are largely shaped by internal working models of attachment across the lifespan, future research may seek to examine defensive functioning through the lens of attachment to inform psychodynamic approaches and defense scales [41]. Future interventions may integrate defenses by considering the maladaptive defenses of children in the face of painful emotions [69] and the effects of disorganized attachment on defensive behaviors in response to environmental threats approaching one’s body [100]. Implementing defense models such as the ADQ-50 and substantiating their effectiveness in various contexts may also be beneficial in tailoring treatments [3]. Further longitudinal studies are needed to assess changes in defense mechanisms and attachment patterns following psychological interventions [5].

To further validate the conclusions about attachment classifications, future studies may seek to implement larger, stratified samples [5,20,79] that control for factors such as psychopathological symptoms [7] and medication [4] as part of the study design. There also remains a gap in understanding the role of gender in parent–child attachment, and future studies may seek to examine how fathers influence children’s ER strategies and attachment styles [7]. Moreover, given the higher prevalence of female psychology providers compared to their male counterparts, additional research may investigate the effects of patient–provider gender discrepancies in psychotherapy, with a focus on how clinical approaches can be tailored to the unique needs of each gender in the therapeutic dyad [68]. As lower income has been correlated with attachment avoidance in women [80], the influence of gender and other sociocultural factors on attachment style should be further studied.

Future studies on regulatory strategies may also aim to integrate more male and elderly participants, as these populations remain understudied in the existing literature [64,101]. There is also a need for further research on attachment and ER patterns that accounts for various other factors, including race [81], socioeconomic status [80], and adverse childhood experiences [102].

## 9. Conclusions

Attachment theory offers an important lens for understanding a patient. Clinicians can find insight into a patient’s attachment classification through a consideration and evaluation of their habitual defenses as manifesting within the clinical encounter. By assessing these interrelated processes, as informed by an accumulation of empirical work, clinicians can more precisely tailor interventions to patients’ unique needs. Integrating an awareness of social context and adversity ensures interventions address not just intrapersonal, but also psychosocial and interpersonal contributors to psychological suffering. This comprehensive clinical approach will likely be effective in promoting adaptive development and functioning, as well as psychological growth throughout the lifespan.

## Figures and Tables

**Table 1:** Data collection and extraction.

Research Question	What links has literature drawn between attachment classification, emotion regulation, and defense mechanisms under the framework of developmental theory?
Databases	EBSCOhost, ProQuest, APA PsycArticles, SAGE, Science Direct, Taylor & Francis Online, Wiley Online
Search terms	Attachment, emotion regulation, psychotherapy, social determinants of health, defense mechanisms
Hand Search	*N* = 103

**Table 2:** Patient attachment styles and correlates with emotion regulation strategies and defense mechanisms.

Attachment Style	Life Stage	ER Strategy	ER Examples	Defense Mechanisms	Defense Mechanism Examples
Secure [7,15,23,24,25,27,28,40,41,42,43]	Infancy	Coregulation; flexible up-/down-regulation	Social referencing; joint attention; protest, then rapid soothing upon reunion; self-soothing (thumb-sucking); gaze alternation	Early regulatory precursors (affiliation/ help-seeking)	Brief distress with rapid settling; proximity seeking, then exploration
	Adolescence/Adulthood	Reappraisal; reflective functioning/ mentalization; appropriate suppression	Emotional suppression paired with expectations of caregiver attunement; reframing	Mature/adaptive operations (e.g., adaptive affiliation, undoing)	Coherent narrative; affect–report concordance; help-seeking; misdeeds followed by reparative actions
Anxious/ Ambivalent/Preoccupied [3,19,20,29,35,37,38,39,41,42,43,68]	Infancy	Hyperactivation	Emotional intensification; difficulty soothing after separation; psychomotor shifts/acting out	Caregiver proximity-seeking	Prolonged protest; reduced exploration; rapid re-escalation
	Adolescence/Adulthood	Hyperactivation; rumination Cognitive reappraisal	Intensification of sadness/worry/anger; hypervigilance to painful cues; reappraisal/suppression to reduce anger	Splitting; projective identification; acting out; passive aggression; reaction formation; undoing	Heightened threat monitoring; black-and-white evaluations; abrupt tone shifts
Avoidant/Dismissing [3,5,17,19,40,41,42,43,44,45,46,47]	Infancy	Deactivation	Minimized outward distress during separation; emotional distancing; psychomotor shifts; abrupt refusal to play	Proto-deactivating organization	Limited proximity bids; muted or indirect signaling
	Adolescence/Adulthood	Deactivation; suppression	Dampened expression of sadness/anxiety/grief; cognitive/behavioral disengagement	Isolation; deflection; suppression	Topic avoidance; flat/incongruent affect; marked hesitation/monotone
Fearful/ Unresolved/Disorganized [7,19,48,49,52,53,54]	(Childhood trauma onset; labeled in adulthood)	Incoherent strategy; suppression	Heightened stress reactivity; prolonged recovery; rapid emotional fluctuations	Splitting; dissociation	Disengagement during distress; contradictory shifts in tone/affect

**Table 3:** Clinician-guided attachment-related assessments.

Assessment Name	Assessment Type	Procedure	Age Group	Constructs Measured
Strange Situation Procedure (SSP) [29,45,77,78]	Observational	Structured caregiver separations/ reunions; coded for infant behavior to classify attachment.	Infants	Attachment classification (secure, avoidant, resistant/ambivalent, disorganized); behavioral ER organization.
Attachment Interview for Adolescents (AIA) [15]	Narrative-based interview	Semi-structured interview on attachment experiences; narratives coded for attachment representations.	Adolescents	Internal attachment models; indicators of explicit/behavioral ER.
Adult Attachment Interview (AAI) [2,11,51,77]	Narrative-based interview	Semi-structured autobiographical interview; coded for state of mind with respect to attachment.	Adolescents, Adults	Attachment representation; indirect indicators of implicit ER/defensive organization.
Adult Attachment Projective (AAP) [2,15,71,72,73]	Narrative-based projective	Narratives to attachment- related drawings; coded for attachment representations.	Adolescents, Adults	Attachment representations; indirect indicators of implicit ER/defenses.
Defense Mechanisms Rating Scales Q-Sort (DMRS-Q) [5,20,25,79]	Observer-coded (Q-sort)	Code defenses from clinical material (e.g., sessions/ transcripts) across maturity levels.	Adults	Defense mechanisms; implicit ER; reflective functioning/ mentalization (related).
Attachment Defenses Questionnaire (ADQ-50) [3]	Self-report	50-item questionnaire indexing defense mechanisms associated with attachment processes.	Adults	Attachment- related defense factors (10-factor structure).

**Table 4:** Clinician-guided responses to attachment styles.

Attachment Style	Clinical Response	Summary
Secure [7,34]	Build on adaptive strengths to support growth in stress; deepen insight.	Leverage strengths; promote resilience.
Anxious/Ambivalent/Preoccupied [36,38]	Stabilize arousal before cognitive work; use reappraisal to interrupt rumination and reliance on suppression.	Downshift arousal; reduce ruminative loops.
Avoidant/Dismissing [5,47]	Increase awareness and tolerance of attachment-related emotion; gently interrupt automatic deactivating strategies (suppression, disengagement, intellectualization) and support safe, graded proximity and dependence.	Broaden affect access and reliance on others while softening deactivation; increase authentic emotional engagement.
Fearful/Unresolved/Disorganized [34,48,49]	Provide structured, trauma-informed education to reduce alienating behavior; prioritize stabilization; consider mentalization-based therapy and DBT to improve emotion identification/clarity and reduce immature defenses.	Safety, stabilization, and trauma-informed skills.

## Data Availability

No new data were created or analyzed in this study. Data sharing is not applicable to this article.
